# Correction: Strawberry sweetness and consumer preference are enhanced by specific volatile compounds

**DOI:** 10.1038/s41438-021-00664-2

**Published:** 2021-10-06

**Authors:** Zhen Fan, Tomas Hasing, Timothy S. Johnson, Drake M. Garner, Michael L. Schwieterman, Christopher R. Barbey, Thomas A. Colquhoun, Charles A. Sims, Marcio F. R. Resende, Vance M. Whitaker

**Affiliations:** 1 Horticultural Sciences Department, University of Florida, IFAS Gulf Coast Research and Education Center, Wimauma, FL USA; 2 Elo Life Systems, Durham, NC USA; 3 Department of Environmental Horticulture and Plant Innovation Center, Institute of Food and Agricultural Sciences, University of Florida, Gainesville, FL USA; 4 Plenty Unlimited, Inc., South San Francisco, CA 94080 USA; 5 Food Science and Human Nutrition Department, University of Florida, Gainesville, FL USA; 6 Horticultural Sciences Department, University of Florida, Gainesville, FL USA

**Keywords:** Plant breeding, Metabolomics

Correction to: *Horticulture Research*

10.1038/s41438-021-00502-5, published online 01 April 2021

Since the publication of this article^1^, the corresponding author discovered that an additional researcher contributed data and should be included as an author. Michael L. Schwieterman (Plenty Unlimited, Inc., South San Francisco, CA 94080, US) has been added as the fifth author.

The original article has been corrected.
