# Supplementary material for: Strawberry sweetness and consumer preference are enhanced by specific volatile compounds
Source: Hortic Res. 2021 Apr 1;8:66. doi: 10.1038/s41438-021-00502-5 (PMC8012349; doi:10.1038/s41438-021-00502-5)
Supplement: Supplementary file 1 — Table S1 [file 41438_2021_502_MOESM1_ESM.docx]

|  | Sweetness | Sourness | Texture | Flavor | Liking |
| --- | --- | --- | --- | --- | --- |
| Sweetness | 1 | 0.41073* | 0.59229* | 0.83810* | 0.71473* |
| Sourness |  | 1 | 0.36337* | 0.55401* | 0.29837* |
| Texture |  |  | 1 | 0.63082* | 0.78274* |
| Flavor |  |  |  | 1 | 0.68778* |
| Liking |  |  |  |  | 1 |

A

*Significant at α = 0.01

B

|  | Sourness | Texture | Flavor | Liking |
| --- | --- | --- | --- | --- |
| Sourness | 1 | 0.16349* | 0.42176* | 0.00754 |
| Texture |  | 1 | 0.30582* | 0.63779* |
| Flavor |  |  | 1 | 0.23266* |
| Liking |  |  |  | 1 |
