# Supplementary figures and images for: Strawberry sweetness and consumer preference are enhanced by specific volatile compounds
Source: Hortic Res. 2021 Apr 1;8:66. doi: 10.1038/s41438-021-00502-5 (PMC8012349; doi:10.1038/s41438-021-00502-5)

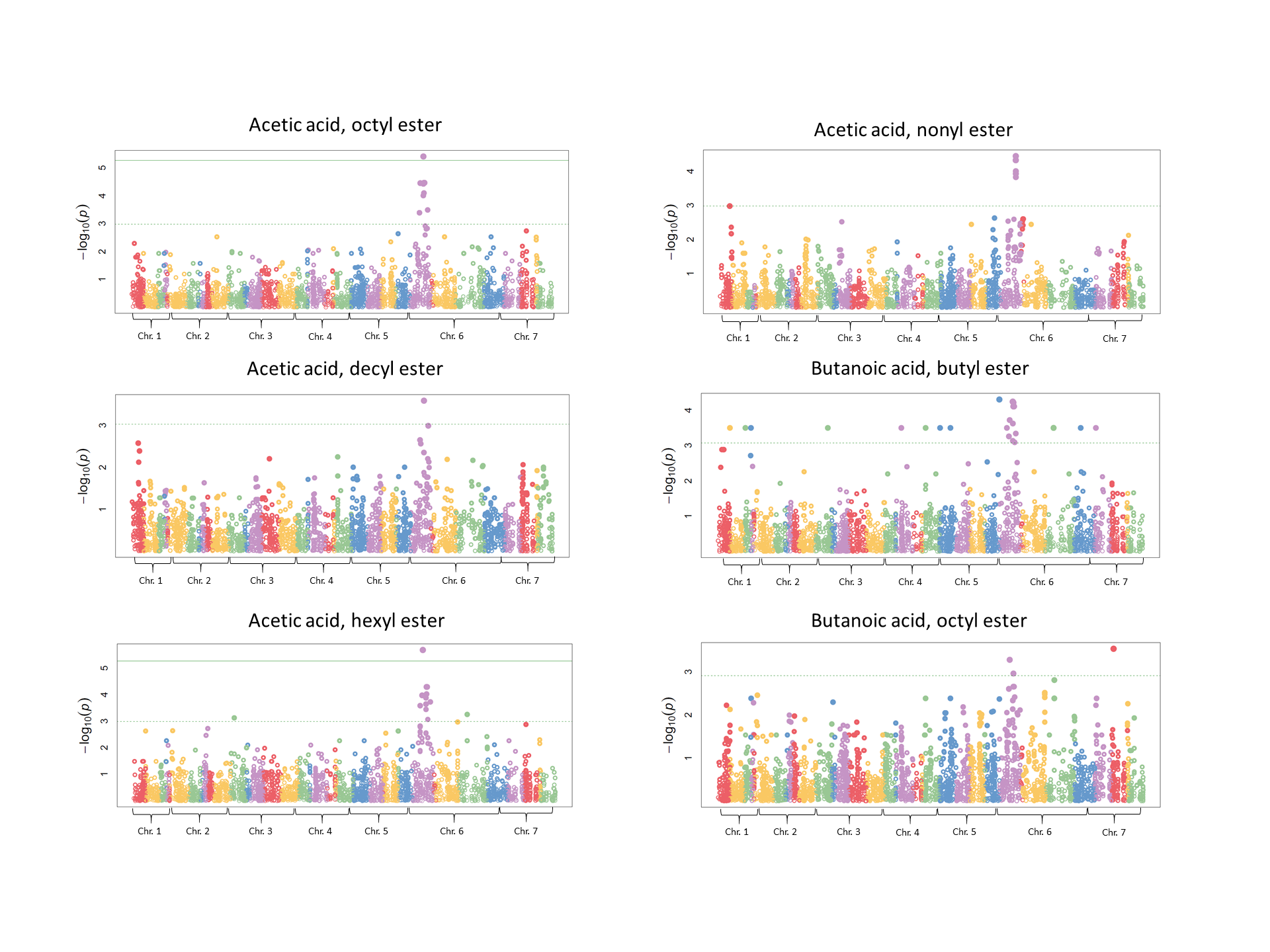

Supplement: Supplementary file 8 — Figure S1 [file 41438_2021_502_MOESM8_ESM.png]

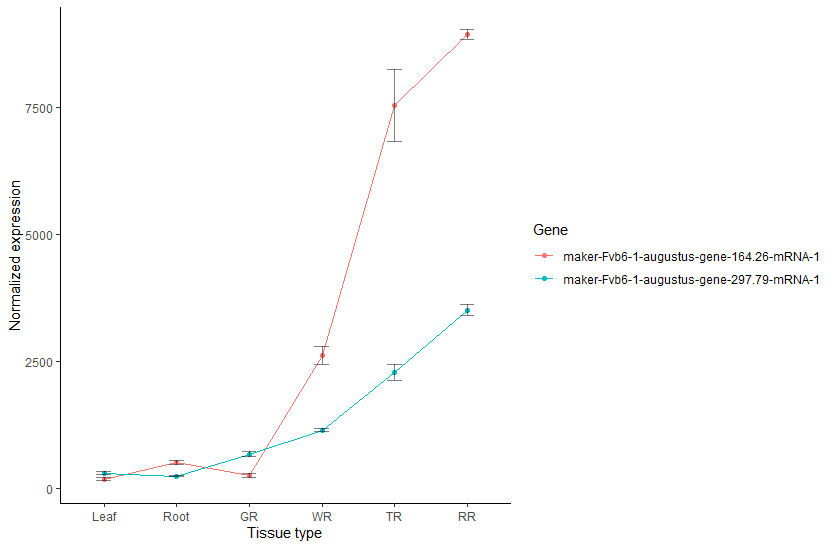

Supplement: Supplementary file 9 — Figure S2 [file 41438_2021_502_MOESM9_ESM.png]
